# Abnormal eye movements in spinocerebellar ataxia type 3

**DOI:** 10.1186/s12883-021-02057-3

**Published:** 2021-01-19

**Authors:** Junyu Lin, Lingyu Zhang, Bei Cao, Qianqian Wei, Ruwei Ou, Yanbing Hou, Xinran Xu, Kuncheng Liu, Xiaojing Gu, Huifang Shang

**Affiliations:** Department of neurology, Laboratory of Neurodegenerative Disorders, Rare Diseases Center, West China Hospital, Sichuan University, Chengdu, Sichuan China

**Keywords:** Spinocerebellar Ataxia type 3, Eye movements, Severity

## Abstract

**Background:**

Abnormal eye movements are common in spinocerebellar ataxias Type 3 (SCA3). We conducted the research to explore the frequency of abnormal eye movements in Chinese patients with SCA3, to compare the demographic and clinical characteristics between SCA3 patients with and without each type of abnormal eye movement, and to explore the correlation between abnormal eye movements and the severity of ataxia.

**Methods:**

Seventy-four patients with SCA3 were enrolled in this cross-sectional study. Six types of abnormal eye movements including impaired smooth pursuit, increased square-wave jerks (SWJ), gaze-evoked nystagmus (GEN), slowing of saccades, saccadic hypo/hypermetria and supranuclear gaze palsy were evaluated by experienced neurologists. The severity of ataxia was evaluated by Scale for the Assessment and Rating of Ataxia (SARA).

**Results:**

The prevalence of impaired smooth pursuit, increased SWJ, GEN, slowing of saccades, saccadic hypo/hypermetria and supranuclear gaze palsy in Chinese SCA3 patients was 28.4, 13.5, 78.4, 41.9, 23.0, and 5.4%, respectively. SCA3 patients with GEN had higher scores of International Cooperative Ataxia Rating Scale (ICARS-IV) and total ICARS, and longer length of CAG repeat than patients without GEN. SCA3 patients with slowing of saccades had a longer disease duration, higher scores of ICARS-I, ICARS-II, total ICARS and SARA than patients without slowing of saccades. SCA3 patients with saccadic hypo/hypermetria had higher scores of ICARS-III, ICARS-IV, and SARA than patients without saccadic hypo/hypermetria. The demographic and clinical characteristics did not differ significantly between SCA3 patients with and without impaired smooth pursuit, increased SWJ, or supranuclear gaze palsy. Multivariate linear regression showed that the number of abnormal eye movements (0–6), disease duration, Hamilton Depression Rating Scale-24 (HDRS-24) score, and CAG repeat length were positively correlated with SARA score, whereas Montreal Cognitive Assessment (MoCA) score was negatively correlated with SARA score in SCA3.

**Conclusions:**

An increased number of abnormal eye movement types correlated with the severity of ataxia in SCA3.

**Supplementary Information:**

The online version contains supplementary material available at 10.1186/s12883-021-02057-3.

## Background

The spinocerebellar ataxias (SCAs) are a genetically heterogeneous group of autosomal dominantly inherited hereditary ataxia characterized by loss of balance, dysdiadochokinesia, and slurred speech. Spinocerebellar ataxias type 3 (SCA3), also known as Machado-Joseph disease, is the most common type of SCAs worldwide caused by CAG expansion of *ATXN3* gene. In China, SCA3 is also the most common subtype of SCAs which accounts for 48–49% of SCA patients [1].

Abnormal eye movements are common in SCAs, including impaired smooth pursuit, increased square-wave jerks (SWJ), gaze-evoked nystagmus (GEN), slowing of saccades, saccadic hypo/hypermetria and supranuclear gaze palsy [2–4]. A few studies have focused on the potential distinguish role of abnormal eye movements among SCA subtypes [2, 5–9]. Based on these studies, abnormal eye movements are frequent in patients with SCA3 [2, 6]. For example, nystagmus has been reported to present in 88% of patients with SCA3 [10]. However, the frequency of each type of abnormal eye movement in Chinese patients with SCA3 have never been studied systematically.

In addition, limited studies have compared the demographic and clinical characteristics between SCA3 patients with and without a certain type of abnormal eye movement, or.

investigated the correlation between abnormal eye movements and the severity of ataxia in patients with SCA3. A study found higher scores of Scale for the Assessment and Rating of Ataxia (SARA) in SCA3 patients with ophthalmoparesis or slowing of saccades [6]. A study observed that oculomotor score of Brief Ataxia Rating Scale (BARA) correlated with disease severity, which was calculated pathogenic by BARA total score minus oculomotor score, in SCA3 patients using univariate correlation analysis [2]. Another study found a positive correlation between SARA score and frequency of SWJ, frequency and amplitude of horizontal GEN and upward saccade latency, and a negative correlation between SARA score and horizontal and upward saccade velocity and accuracy in SCA3 patients using univariate correlation analysis [11]. However, no multivariate analysis has been conducted to confirm the association between abnormal eye movements and the severity of ataxia in SCA3.

Therefore, in the current study, we aimed to detect the frequency of each type of abnormal eye movement type in Chinese patients with SCA3, to compare the demographic and clinical characteristics between SCA3 patients with and without each type of abnormal eye movement, and to investigate the correlation between abnormal eye movements and the severity of ataxia using multivariate analysis model.

## Methods

### Patients evaluation

This study was conducted in agreement with the Ethics Committee of West China Hospital of Sichuan University. All recruited participants provided a written informed consent. A total of 74 genetically confirmed SCA3 patients (39 male) were consecutively recruited from the Department of Neurology, West China Hospital of Sichuan University between August 2015 and January 2020.

In the current study, we performed a cross-sectional study. After testing for trinucleotide repeat expansions of genes causing *SCA1*, *SCA2*, *SCA3*, *SCA6* and *SCA7* using short tandem repeat (STR) analysis, all patients received a genetically confirmed diagnosis of SCA3. The CAG repeat lengths of the expanded allele were collected. All the patients underwent a face-to-face interview in our department. The following demographic and clinical data were collected: sex, age, weight, height, educational years, age of onset, and disease duration. Body-mass index (BMI) was calculated by body weight (kg) divided by heights squared (m^2^). Global cognitive function was assessed using Montreal Cognitive Assessment (MoCA) [12]. Hamilton Depression Rating Scale-24 (HDRS-24) was used to screen depression [13]. Hamilton Anxiety Rating Scale (HARS) was used to screen anxiety [14]. Epworth Sleepiness Scale (ESS) was used to screen excessive daytime sleepiness [15]. Pittsburgh sleep quality index (PSQI) was used to screen sleep problems [16].

Eye movement abnormalities were evaluated using accepted bedside techniques [17] by neurologists who were experienced in movement disorders, including impaired smooth pursuit, increased SWJ, GEN, slowing of saccades, saccadic hypo/hypermetria and supranuclear gaze palsy. Impaired smooth pursuit, GEN, slowing of saccades, and saccadic hypo/hypermetria were evaluated following the International Cooperative Ataxia Rating Scale (ICARS) procedure [18]. Pursuit was evaluated both in horizontal and vertical planes. SWJ were detected during central fixation, and increased SWJ were defined as SWJ ≥ 10 per minute [19]. Saccades were evaluated both in horizontal and vertical planes for speed and accuracy. GEN was evaluated in eccentric gaze both in horizontal and vertical planes. Supranuclear gaze palsy was evaluated both in horizontal and vertical planes. Vision loss was also collected.

Ataxia severity was assessed using ICARS [18] and SARA [20]. ICARS is constituted by four symptomatologic compartments: postural and stance disorders, limb ataxia, dysarthria and oculomotor disorders [18]. SARA is composed of eight items: gait, stance, sitting, speech disturbance, finger chase, nose-finger test, fast alternating hand movements and heel-shin slide. SARA has been proved to be a reliable and valid clinical scale measuring the severity of ataxia [20]. In addition, oculomotor disorders are not included in the items of SARA, which is appropriate to avoid multicollinearity. Therefore, we chose SARA as the dependent variable to reflect the severity of ataxia in the multiple linear regression model.

### Statistical analysis

All continuous variables were presented as the mean ± standard deviation and all categorical variables were presented as numbers or percentages. The demographic and clinical characteristics were compared between SCA3 patients with and without each type of abnormal eye movement (impaired smooth pursuit, increased SWJ, GEN, slowing of saccades, saccadic hypo/hypermetria and supranuclear gaze palsy) respectively. Continuous variables were compared using student’s t-test if they accorded with normal distribution, and Mann-Whitney U test if they did not accord with normal distribution, and categorical variables using Chi-squared test or Fisher’s exact test. ICARS-I, ICARS-II, ICARS-III, ICARS-IV, total ICARS scores, and SARA scores were compared using analyses of covariance (ANCOVA) with adjustment for age and disease duration. MoCA scores were compared using ANCOVA with adjustment for age and educational years. The *p*-values were false discovery rate (FDR)-corrected for multiple comparisons to avoid false positive significances following the Benjamini-Hochberg (BH) procedure. Spearman’s rank correlation analyses were performed to assess the correlation between SARA scores with other variables, including sex, age, disease duration, BMI, CAG repeat length, HDRS-24 score, HARS score, PSQI score, MoCA score, and number of type of abnormal eye movement (0–6, impaired smooth pursuit, increased SWJ, GEN, slowing of saccades, saccadic hypo/hypermetria and supranuclear gaze palsy). Age, disease duration, CAG repeat length, and other variables with a *P* value less than 0.10 were entered into the next multivariate linear regression model to predict the severity of ataxia (SARA score) as covariables. Multicollinearity was diagnosed using tolerance and variance inflation factor (VIF). Tolerance less than 0.2 or VIF greater than 5 suggested the existence of multicollinearity. All analyses were performed using the Statistical Package for the Social Sciences (SPSS) version 22.0. Two-tailed *p* values of < 0.05 were considered statistically significant.

## Results

**Fig. 1:**
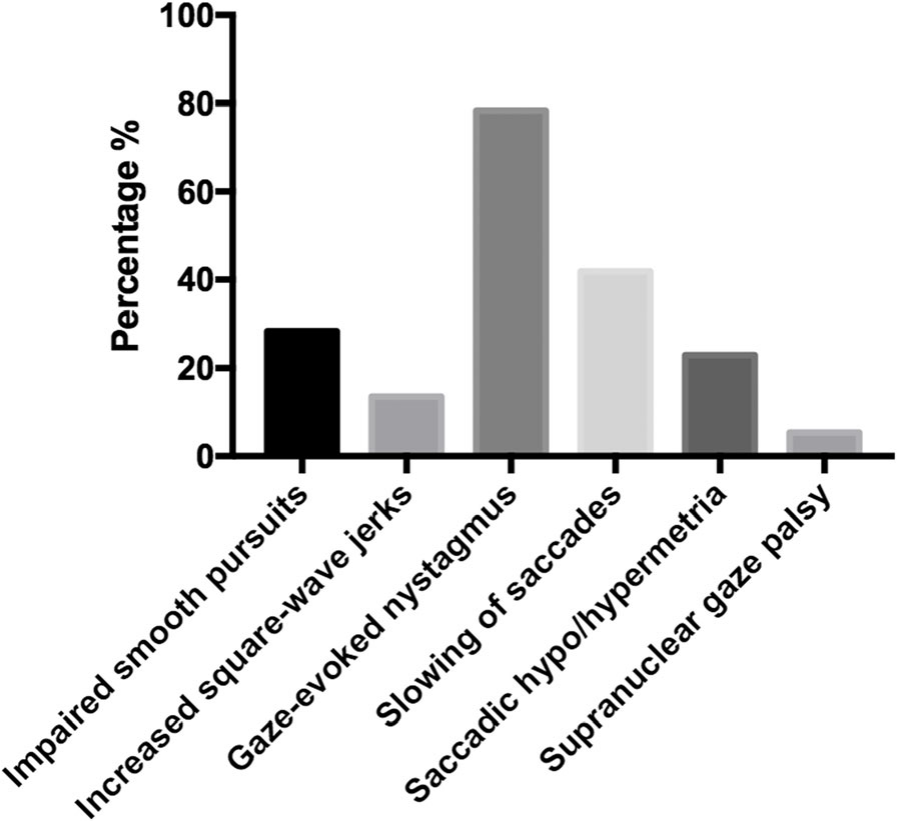
Frequency of each type of abnormal eye movement in SCA3. X-axis demonstrates the type of each abnormal eye movement, and Y-axia shows the frequency of each abnormal eye movement. The frequency of impaired smooth pursuit, increased square-wave jer ks, gaze-evoked nystagmus, slowing of saccades, saccadic hypo/hypermetria and supranuclear gaze palsy was 28.4, 13.5, 78.4, 41.9, 23.0, and 5.4% respectively


A total of 74 SCA3 patients (39 males and 35 females) with mean disease duration of 6.25 ± 6.24 years were recruited in the current study (Table 1). Up to 90.5% of the SCA3 patients had at least one type of abnormal eye movement (see Video). The frequency of each type of abnormal eye movement such as impaired smooth pursuit, increased SWJ, GEN, slowing of saccades, saccadic hypo/hypermetria and supranuclear gaze palsy was 28.4, 13.5, 78.4, 41.9, 23.0 and 5.4% respectively (Fig. 1).

**Table 1:** Demographic and clinical features of the recruited SCA3 patients

Variable	Cross-sectional study
	(*n* = 74)
Sex (male,%)	39 (52.7%)
Mean age (years)	45.04 ± 11.97
Age of onset (years)	38.79 ± 12.53
Disease duration (years)	6.25 ± 6.24
Educational year	10.53 ± 4.52
BMI	23.69 ± 21.46
Hyperreflexia (%)	33 (44.6%)
Vision loss	24 (32.4%)
ICARS-I	12.27 ± 6.71
ICARS-II	15.69 ± 8.97
ICARS-III	1.95 ± 1.53
ICARS-IV	2.24 ± 1.40
Total ICARS score	32.15 ± 16.31
SARA score	11.01 ± 5.73
MoCA score	23.58 ± 4.68
HDRS-24 score	8.80 ± 8.92
HARS score	6.82 ± 7.04
ESS score	5.41 ± 4.67
PSQI score	7.23 ± 4.46
CAG repeat length	66.07 ± 11.15

*SCA3* spinocerebellar ataxia 3; *BMI* body mass index; *ICARS* International Cooperative Ataxia Rating Scale; *SARA* Scale for the Assessment and Rating of Ataxia; *MoCA*, Montreal Cognitive Assessment; *HDRS-24* Hamilton Depression Scale; *HARS* Hamilton Anxiety Scale; *ESS* Epworth Sleepiness Scale; *PSQI* Pittsburgh sleep quality index

**Additional file 1.** Segment 1. This patient demonstrates impaired smooth pursuit; Segment 2. This patient demonstrates increased square-wave jerks; Segment 3. This patient demonstrates gaze-evoked nystagmus; Segment 4. This patient demonstrates slowing of saccades; Segment 5. This patient demonstrates saccadic dysmetria; Segment 6. This patient demonstrates vertical supranuclear gaze palsy.

Demographic and clinical characteristics of the SCA3 patients with and without each abnormal eye movement (impaired smooth pursuit, increased SWJ, GEN, slowing of saccades, saccadic hypo/hypermetria and supranuclear gaze palsy) are shown in Table 2. SCA3 patients with GEN had high scores of ICARS-IV and total ICARS, and longer length of CAG repeat than patients without GEN. SCA3 patients with slowing of saccades had a longer disease duration, a high score of ICARS-I, ICARS-II, total ICARS and SARA than patients without slowing of saccades. SCA3 patients with saccadic hypo/hypermetria had a high score of ICARS-III, ICARS-IV, and SARA than patients without saccadic hypo/hypermetria. The demographic and clinical characteristics did not differ significantly between SCA3 patients with and without impaired smooth pursuit, increased SWJ, or supranuclear gaze palsy.

**Table 2:** Demographic and clinical features of the SCA3 patients with and without each type of abnormal eye movement

	Impaired smooth pursuits (horizontal or vertical)			Increased square-wave jerks			Gaze-evoked nystagmus (horizontal or vertical)		
	Yes (*n* = 21)	No (*n* = 53)	P value	Yes (*n* = 10)	No (*n* = 64)	P value	Yes (*n* = 58)	No (*n* = 16)	P value
Sex (male,%)^c^	10 (47.6%)	29 (54.7%)	0.693	2 (20.0%)	37 (57.8%)	0.727	31 (53.4%)	8 (50.0%)	0.807
Mean age (years)^a^	45.05 ± 12.47	45.04 ± 11.90	0.919	43.20 ± 9.81	45.33 ± 12.32	0.789	43.81 ± 11.66	49.50 ± 12.40	0.215
Age of onset (years)^a^	37.38 ± 11.78	39.35 ± 12.88	0.693	37.20 ± 9.40	39.04 ± 12.99	0.789	38.03 ± 11.62	41.56 ± 15.49	0.222
Disease duration (years)^b^	7.67 ± 5.30	5.69 ± 6.54	0.200	6.00 ± 5.54	6.29 ± 6.38	0.885	5.78 ± 4.10	7.94 ± 11.04	0.760
Educational year^b^	10.00 ± 4.04	10.74 ± 4.72	0.688	9.20 ± 3.36	10.73 ± 4.66	0.789	11.03 ± 4.57	8.69 ± 3.93	0.089
BMI^b^	20.81 ± 2.63	24.83 ± 25.28	0.693	21.15 ± 3.38	24.08 ± 23.04	0.885	21.07 ± 2.99	33.16 ± 45.67	0.241
Hyperreflexia^c^	10 (47.6%)	23 (43.4%)	0.824	6 (60%)	27 (42.2%)	0.789	30 (51.7%)	3 (18.8%)	0.067
Vision loss^c^	9 (42.9%)	15 (28.3%)	0.429	6 (60.0%)	18 (28.1%)	0.727	22 (37.9%)	2 (12.5%)	0.133
ICARS-I^d^	14.91 ± 7.25	11.23 ± 6.24	0.223	11.20 ± 4.37	12.44 ± 7.01	0.789	12.93 ± 6.30	9.88 ± 7.74	0.055
ICARS-II^d^	19.38 ± 10.12	14.23 ± 8.12	0.165	13.00 ± 8.54	16.11 ± 9.03	0.789	16.38 ± 8.75	13.19 ± 9.59	0.067
ICARS-III^d^	2.43 ± 1.78	1.76 ± 1.40	0.398	1.50 ± 1.35	2.02 ± 1.56	0.789	1.95 ± 1.48	1.94 ± 1.77	0.722
ICARS-IV^d^	3.05 ± 1.43	1.93 ± 1.27	0.060	2.90 ± 1.60	2.14 ± 1.36	0.727	2.55 ± 1.27	1.13 ± 1.31	0.020*
Total ICARS score^d^	39.76 ± 18.04	29.13 ± 14.68	0.113	28.60 ± 14.14	32.70 ± 16.65	0.789	33.81 ± 15.39	26.13 ± 18.58	0.040*
SARA score^d^	13.79 ± 6.17	9.91 ± 5.21	0.113	9.70 ± 3.74	11.21 ± 5.98	0.789	11.41 ± 5.35	9.56 ± 6.94	0.133
MoCA score^e^	22.28 ± 5.33	24.06 ± 4.36	0.398	24.00 ± 2.00	23.52 ± 4.98	0.789	24.28 ± 4.20	21.06 ± 5.58	0.241
HDRS-24 score^b^	9.00 ± 8.16	8.72 ± 9.28	0.683	6.50 ± 3.75	9.16 ± 9.45	0.885	9.55 ± 9.60	6.06 ± 5.17	0.396
HARS score^b^	6.76 ± 4.79	6.85 ± 7.80	0.429	4.80 ± 4.42	7.14 ± 7.34	0.789	7.66 ± 7.60	3.81 ± 3.08	0.215
ESS score^b^	5.33 ± 5.03	5.43 ± 4.57	0.867	6.30 ± 5.60	5.27 ± 4.55	0.789	5.59 ± 4.67	4.75 ± 4.77	0.608
PSQI score^b^	8.33 ± 4.09	6.79 ± 4.56	0.280	6.00 ± 2.98	7.42 ± 4.64	0.789	7.36 ± 4.57	6.75 ± 4.14	0.722
CAG repeat length^b^	64.19 ± 13.22	66.81 ± 10.27	0.693	69.70 ± 3.23	65.50 ± 11.84	0.789	68.48 ± 7.33	57.31 ± 17.22	0.040*
	Slowing of saccades (horizontal or vertical)			Saccadic hypo/hypermetria (horizontal or vertical)			Supranuclear gaze palsy (horizontal or vertical)		
	Yes (*n* = 31)	No (*n* = 43)	P value	Yes (*n* = 17)	No (*n* = 57)	P value	Yes (n = 4)	No (*n* = 70)	P value
Sex (male,%)^c^	19 (61.3%)	20 (46.5%)	0.523	13 (76.5%)	26 (45.6%)	0.100	3 (75.0%)	36 (51.4%)	0.840
Mean age (years)^a^	45.68 ± 10.54	44.58 ± 13.01	0.665	45.00 ± 12.23	45.05 ± 12.01	0.923	39.00 ± 14.72	45.39 ± 11.83	0.775
Age of onset (years)^a^	38.58 ± 11.34	38.94 ± 13.45	0.978	38.18 ± 12.31	38.97 ± 12.40	0.923	31.50 ± 13.63	39.21 ± 12.44	0.740
Disease duration (years)^b^	7.10 ± 4.17	5.64 ± 7.37	0.047*	6.82 ± 5.09	6.08 ± 6.58	0.620	7.50 ± 8.96	6.18 ± 6.13	0.893
Educational year^b^	11.03 ± 4.14	10.16 ± 4.79	0.665	10.06 ± 5.27	10.67 ± 4.31	0.926	9.50 ± 4.12	10.59 ± 4.56	0.775
BMI^b^	20.85 ± 2.61	25.74 ± 28.03	0.665	20.32 ± 2.74	24.69 ± 24.37	0.442	21.65 ± 1.94	23.80 ± 22.07	0.791
Hyperreflexia^c^	16 (51.6%)	17 (39.5%)	0.604	9 (52.9%)	24 (42.1%)	0.662	3 (75.0%)	30 (42.9%)	0.775
Vision loss^c^	8 (25.8%)	16 (37.2%)	0.523	3 (17.6%)	21 (36.8%)	0.345	1 (25.0%)	23 (32.9%)	1.000
ICARS-I^d^	14.74 ± 6.50	10.49 ± 6.34	0.048*	14.71 ± 5.58	11.54 ± 6.88	0.260	10.50 ± 5.92	12.37 ± 6.77	0.775
ICARS-II^d^	18.97 ± 8.91	13.33 ± 8.34	0.048*	18.88 ± 7.88	14.74 ± 9.12	0.260	19.25 ± 9.46	15.49 ± 8.97	0.740
ICARS-III^d^	2.19 ± 1.49	1.77 ± 1.56	0.665	2.94 ± 1.03	1.65 ± 1.54	0.020*	3.00 ± 1.15	1.89 ± 1.54	0.740
ICARS-IV^d^	2.61 ± 1.45	1.98 ± 1.32	0.247	3.24 ± 1.35	1.95 ± 1.29	0.020*	3.25 ± 1.89	2.19 ± 1.37	0.740
Total ICARS score^d^	38.52 ± 15.25	27.56 ± 15.64	0.047*	39.77 ± 13.00	29.88 ± 16.60	0.100	36.00 ± 17.57	31.93 ± 16.34	0.775
SARA score^d^	13.39 ± 5.27	9.29 ± 5.48	0.047*	14.32 ± 4.54	10.02 ± 5.71	0.033*	12.50 ± 4.65	10.92 ± 5.80	0.775
MoCA score^e^	23.03 ± 4.56	23.98 ± 4.78	0.257	22.29 ± 4.93	23.97 ± 4.58	0.371	21.25 ± 2.99	23.71 ± 4.74	0.740
HDRS-24 score^b^	9.36 ± 9.86	8.40 ± 8.28	0.665	7.47 ± 3.92	9.13 ± 9.93	0.804	13.75 ± 7.14	8.51 ± 8.97	0.740
HARS score^b^	7.10 ± 7.52	6.63 ± 6.76	0.751	6.41 ± 4.06	6.95 ± 7.74	0.620	7.00 ± 4.97	6.81 ± 7.17	0.775
ESS score^b^	5.29 ± 4.97	5.49 ± 4.50	0.751	5.41 ± 4.05	5.40 ± 4.88	0.923	3.00 ± 2.94	5.54 ± 4.73	0.775
PSQI score^b^	6.84 ± 4.58	7.51 ± 4.40	0.665	6.94 ± 4.72	7.32 ± 4.42	0.827	7.50 ± 2.52	7.21 ± 4.56	0.840
CAG repeat length^b^	65.65 ± 11.37	66.37 ± 11.12	0.691	65.29 ± 12.99	66.30 ± 10.66	0.987	48.50 ± 19.09	67.07 ± 9.84	0.740

*SCA3* spinocerebellar ataxia 3; *BMI* body mass index; *MoCA* Montreal Cognitive Assessment; *ICARS* International Cooperative Ataxia Rating Scale; *SARA* Scale for the Assessment and Rating of Ataxia; *MoCA*, Montreal Cognitive Assessment; *HDRS-24* Hamilton Depression Scale; *HARS* Hamilton Anxiety Scale; *ESS* Epworth Sleepiness Scale; *PSQI* Pittsburgh sleep quality index

* Significant difference after false discovery rate (FDR) correction for multiple comparisons

^a^ Student’s t-test; ^b^ Mann-Whitney U test; ^c^ Chi-squared test or Fisher’s exact test; ^d^Analyses of covariance (ANCOVA) with adjustment for age and disease duration; ^e^ANCOVA with adjustment for age and educational years

**Fig. 2:**
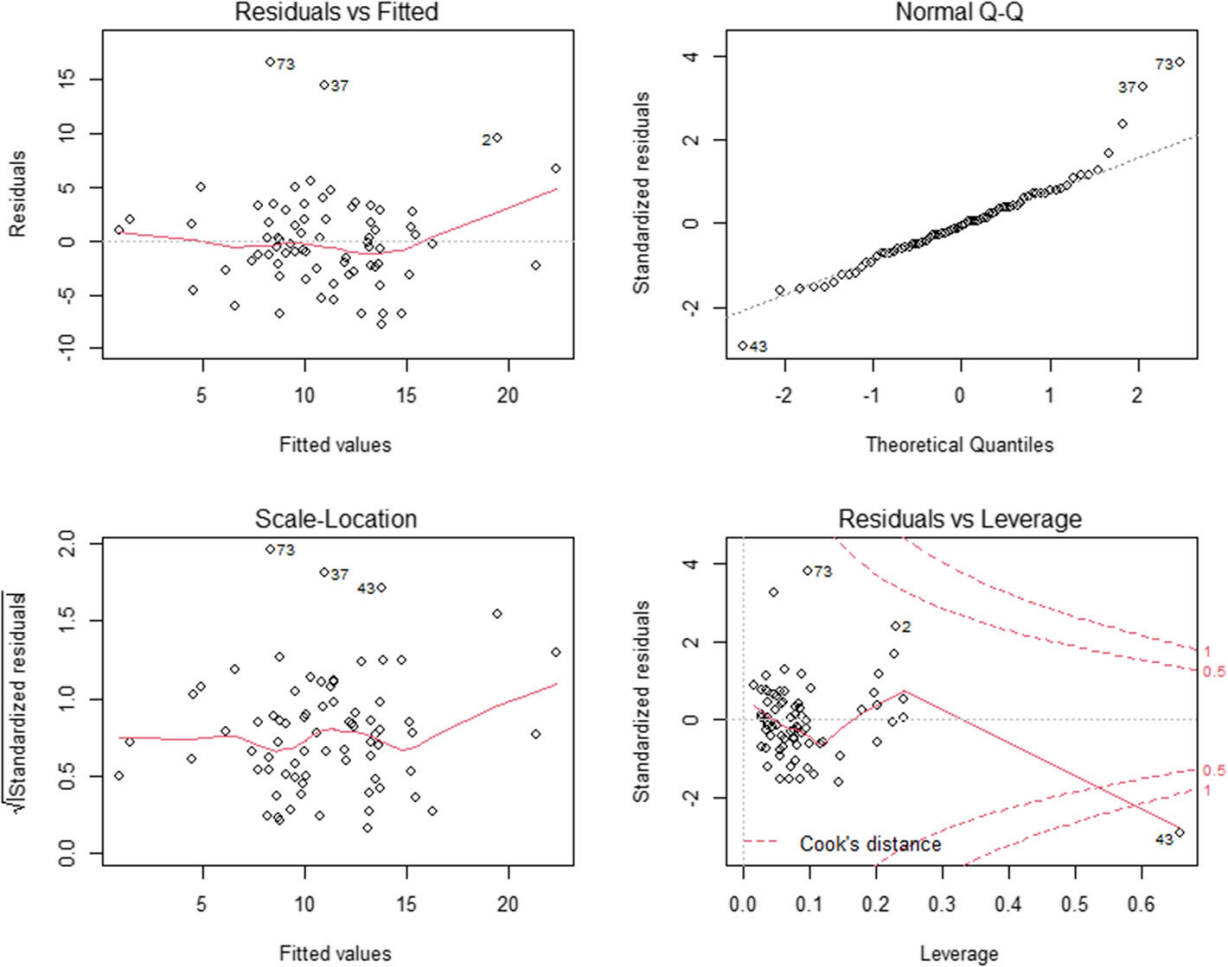
Hypothesis testing for the multivariate linear regression model diagnosis. 1) Residuals vs Fitted figure (upper left) was used to explore the linearity between fitted values and residuals. Data points were uniformly distributed on the both side of y = 0 and the line presented a stationary curve without obvious shape feature, which suggested the linearity was good. 2) Normal Q-Q figure (upper right) was used to examine the normality of standardized residuals. Data points were arranged in diagonal lines, tending to a straight line, and passing diagonally through it, which indicated the residuals were normally distributed. 3) Square root of standardized residuals and fitted figure (lower left) was used to examine the homoscedasticity of standardized residuals. Data points were uniformly distributed on the both side of y = 0 and the line presented a stationary curve without obvious shape feature, which suggested that the residual values of all predicted dependent variables are approximately equal. 4) Standardized residuals vs leverage figure (lower right) was used to check the outlier. There were no significant outliers that can affect the regression results in this model

**Fig. 3:**
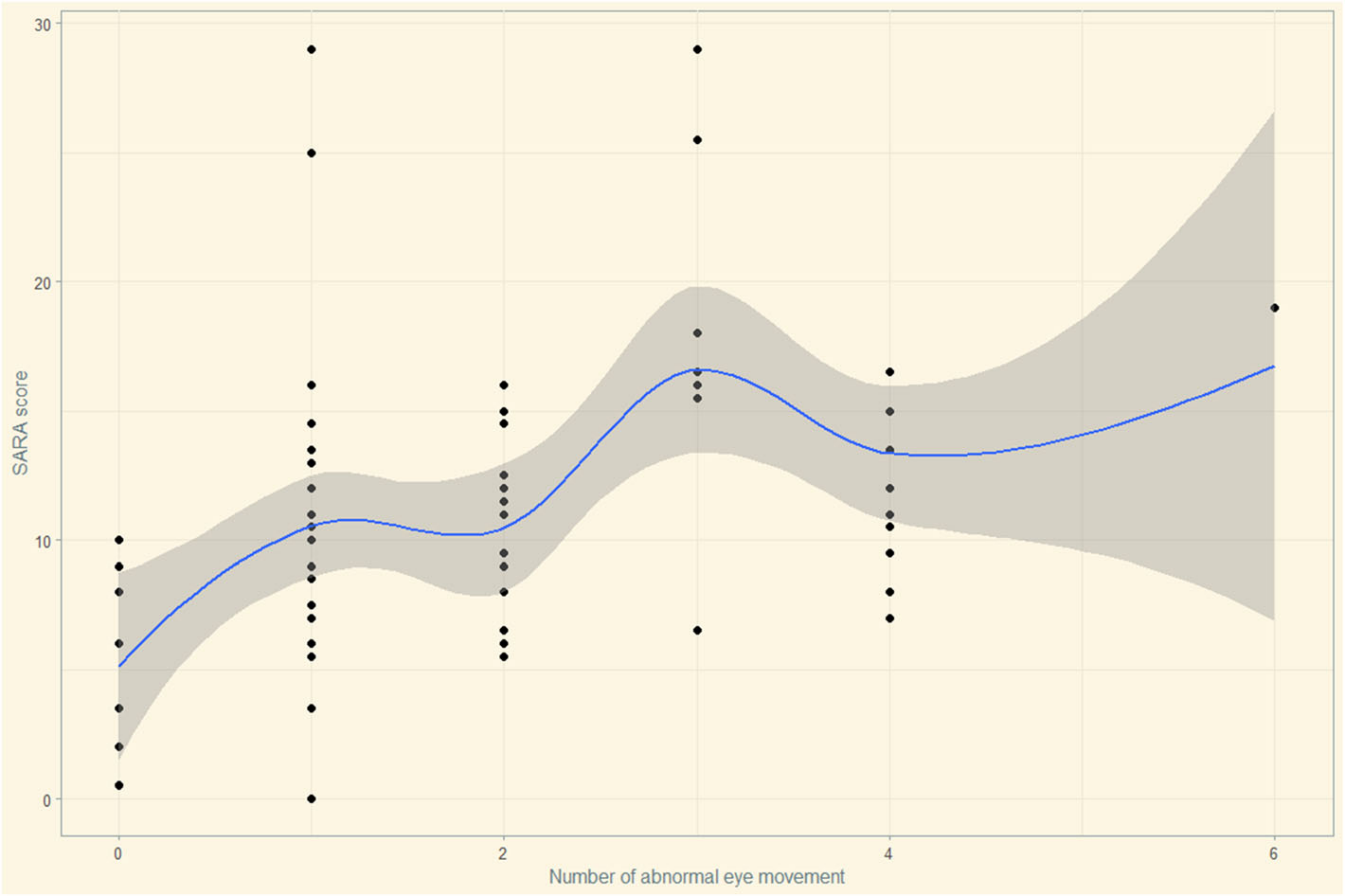
Scatter plots between SARA scores and the numbers of abnormal eye movement. X-axis represents the numbers of abnormal eye movement, and Y-axia represents the SARA scores

Spearman’s rank correlation analyses showed that the SARA score was positively correlated with age (r = 0.205, *p* = 0.080), disease duration (r = 0.466, *p* < 0.001), HDRS-24 score (r = 0.234, *p* = 0.045), and number of abnormal eye movement (r = 0.435, p < 0.001), and negatively correlated with MoCA score (r = − 0.266, *p* = 0.022). To investigate the correlation between abnormal eye movements and the severity of ataxia, we performed a multivariate stepwise linear regression analysis. Total SARA score was used to represent the severity of ataxia and acted as the dependent variable, while the number of type of abnormal eye movement (0–6, impaired smooth pursuit, increased SWJ, GEN, slowing of saccades, saccadic hypo/hypermetria and supranuclear gaze palsy) as the independent variable. Other covariables including age, disease duration, HDRS-24 score, MoCA score and CAG repeat length. All independent variables’ tolerance was less than 0.2 and VIF was greater than 5, suggesting there was no multicollinearity in the model. The final model showed the number of type of abnormal eye movement (impaired smooth pursuit, increased SWJ, GEN, slowing of saccades, saccadic hypo/hypermetria and supranuclear gaze palsy) besides disease duration, HDRS-24 score, and CAG repeat length were positively correlated with the severity of ataxia in SCA, whereas MoCA score was negatively correlated with the severity of ataxia in SCA3 (Table 3). The residual plots of the multivariate linear regression model were shown in Fig. 2. Scatter plots between SARA scores and the numbers of abnormal eye movement were drawn to demonstrate the relationship visually (Fig. 3).

**Table 3:** Multivariate stepwise linear regression analysis of the total SARA score in patients with SCA3

Variable	Standardised regression coefficient	Standard error	P value
Disease duration	0.328	0.089	0.001*
HDRS-24 score	0.215	0.062	0.030*
CAG repeat length	0.243	0.050	0.015*
Number of abnormal eye movement	0.310	0.409	0.002*
MoCA score	−0.267	0.120	0.008*

*SARA* Scale for the Assessment and Rating of Ataxia; *SCA3* spinocerebellar ataxia 3; *HDRS-24* Hamilton Depression Scale; *MoCA* Montreal Cognitive Assessment

* Significant difference. *P* value was calculated by a multivariate stepwise linear regression analysis, with age, disease duration, SARA score, CAG repeat length, number of abnormal eye movement, and HDRS-24 score were included as co-variables

## Discussion

In the current study, we observed that GEN was the most common type of abnormal eye movement in Chinese SCA3 patients, followed by slowing of saccades, impaired smooth pursuit, saccadic hypo/hypermetria, increased square-wave jerks, and supranuclear gaze palsy. GEN has been reported to be the most common type of abnormal eye movement in SCA3 by many previous studies [3, 6, 8, 10], whereas supranuclear gaze palsy has been reported uncommon in SCA3 [2], which was in consistent with the current study. The frequency of impaired smooth pursuit detected in the current study was lower than previous reports [2, 3, 6], probably due to the lack of sensitivity of bedside evaluation.

The correlation between CAG repeat numbers and abnormal eye movement remained uncertain. A previous study detected that although not reaching statistical significance, SCA3 patients with GEN had a tendency to have a longer CAG repeat than patients without GEN [10]. In the current study, we found a longer CAG repeat in SCA3 patients with GEN than patients without, while no difference was found in patients with other types of abnormal eye movement. However, another study revealed longer CAG repeats in SCA3 patients with vertical supranuclear gaze palsy and slowing of saccades [6].

The cerebellum has been considered to play an important role in abnormal eye movements [21–23]. The oculomotor sites of the cerebellum (including flocculonodular lobe, uvula, dorsal oculomotor vermis (OMV) and the caudal fastigial nuclei (CFN)) are integrated into the oculomotor circuits that enable normal eye movement [24]. The lesions in the flocculonodular lobe could lead to pursuit disturbances and GEN in monkeys [25]. The OMV (lobule VII and a part of folium VIc) and the CFN seem to be crucial in controlling of saccadic accuracy and smooth pursuit [26–28]. The cerebellum also plays a role in fixation stability, impairment of which would lead to increased SWJ [29]. A pathoanatomic study identified that nearly all of the cerebellar oculomotor structures underwent neurodegeneration in SCA3 patients [30]. The positive correlation between the number of type of abnormal eye movement and the severity of ataxia observed in the current study clinically confirmed the view that neurodegeneration of the cerebellum oculomotor sites contributes to the occurrence of abnormal eye movements, and that the number of type of abnormal eye movement might act as a biomarker to reflect the degree of cerebellum neurodegeneration.

The positive correlation between the HDRS-24 score and the severity of ataxia in patients with SCA3 detected in the current study was in line with previous cross-sectional [31] and longitudinal [32] studies, which confirmed the effects of depression on ataxia progression in patients with SCA3. In addition, the negative correlation between the MoCA score and the severity of ataxia was also in agreement with previous studies [33], indicating that the cognition status can also be an indicator of motor deterioration in patients with SCA3.

This is the first study to investigate the correlation between abnormal eye movements and the severity of ataxia in SCA3 patients using multivariate analysis model. The findings indicated that abnormal eye movements could reflect the degree of cerebellum neurodegeneration in patients with SCA3, making it important to assess abnormal eye movements in SCA3 patients in clinical practice. However, several limitations should be acknowledged in the current study. A major limitation was the lack of electro-oculography or video-oculography to assess abnormal eye movements objectively. However, they were assessed by neurologists who were experienced in movement disorders using accepted bedside techniques described before instead [17]. The second limitation was the lack of head impulse responses date, which are characteristically impaired in SCA3 and may correlate with disease severity [34]. The third limitation was that we only conducted a cross-sectional study, which could only offer a correlation rather than causality. Futher prospective studies are needed to explore whether abnormal eye movements could predict disease progression in SCA3.

## Conclusions

In conclusion, our study confirmed the positive association between abnormal eye movements and the severity of ataxia in SCA3 patients, which emphasize the importance of clinical assessment of abnormal eye movements in patients with SCA3.

## Data Availability

The datasets used and/or analysed during the current study available from the corresponding author on reasonable request.

## Abbreviations

SCA3: Spinocerebellar ataxias Type 3
GEN: Gaze-evoked nystagmus
SARA: Scale for the Assessment and Rating of Ataxia
ICARS: International Cooperative Ataxia Rating Scale
HDRS-24: Hamilton Depression Rating Scale-24
MoCA: Montreal Cognitive Assessment
SWJ: Square-wave jerks
BARA: Brief Ataxia Rating Scale
STR: Short tandem repeat
BMI: Body-mass index
HARS: Hamilton Anxiety Rating Scale
ESS: Epworth Sleepiness Scale
PSQI: Pittsburgh sleep quality index
ANCOVA: Analyses of covariance
FDR: False discovery rate
VIF: Variance inflation factor
SPSS: Statistical Package for the Social Sciences
OMV: Oculomotor vermis
CFN: Caudal fastigial nuclei
